# CyGate Provides a Robust Solution for Automatic Gating of Single Cell Cytometry Data

**DOI:** 10.1021/acs.analchem.3c03006

**Published:** 2023-11-09

**Authors:** Seungjin Na, Yujin Choo, Tae Hyun Yoon, Eunok Paek

**Affiliations:** † Institute for Artificial Intelligence Research, Hanyang University, Seoul 04763, Republic of Korea; ‡ Department of Computer Science, Hanyang University, Seoul 04763, Republic of Korea; § Department of Artificial Intelligence, Hanyang University, Seoul 04763, Republic of Korea; ∥ Department of Chemistry, College of Natural Sciences, Hanyang University, Seoul 04763, Republic of Korea; ⊥ Institute of Next Generation Material Design, Hanyang University, Seoul 04763, Republic of Korea; # Yoon Idea Lab Co., Ltd., Seoul 04763, Republic of Korea

## Abstract

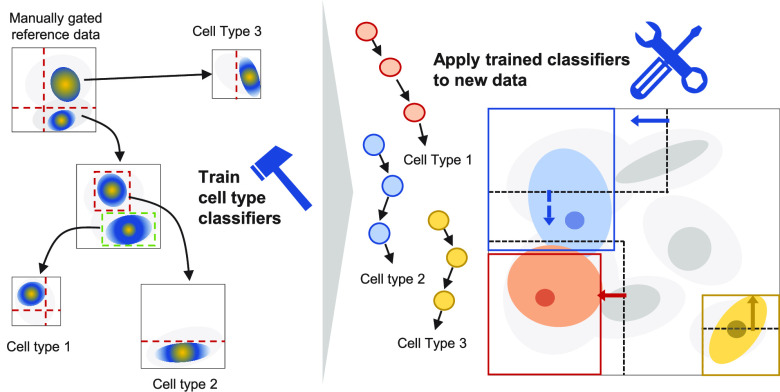

To gain a better understanding of the complex human immune system, it is necessary to measure and interpret numerous cellular protein expressions at the single cell level. Mass cytometry is a relatively new technology that offers unprecedented information about the protein expression of a single cell. Conversely, the analysis of high-dimensional and multiparametric mass cytometric data sets presents a new computational challenge. For instance, conventional “manual gating” analysis was inefficient and unreliable for multiparametric phenotyping of the heterogeneous immune cellular system; consequently, automated methods have been developed to address the high dimensionality of mass cytometry data and enhance the reproducibility of the analysis. Here, we present CyGate, a semiautomated method for classifying single cells into their respective cell types. CyGate learns a gating strategy from a reference data set, trains a model for cell classification, and then automatically analyzes additional data sets using the trained model. CyGate also supports the machine learning framework for the classification of “ungated” cells, which are typically disregarded by automated methods. CyGate’s utility was demonstrated by its high performance in cell type classification and the lowest generalization error on various public data sets when compared to the state-of-the-art semiautomated methods. Notably, CyGate had the shortest execution time, allowing it to scale with a growing number of samples. CyGate is available at https://github.com/seungjinna/cygate.

## Introduction

The human immune system has a diverse range of cell types and signaling molecules, and its responses to external stimuli are known to vary widely. To comprehend such a complex immune system, it is necessary to measure the expression of multiple cellular proteins at the single cell level. Mass cytometry, cytometry by time-of-flight (CyTOF), is an emerging technology for multiparametric, high-dimensional single cell analysis of complex biological systems.^1,2^ In mass cytometry, multiple antibodies tagged with different metal ions bind to each cell and are assessed using time-of-flight mass spectrometry, enabling the simultaneous measurements of over 40 cellular protein markers for millions of cells.^3,4^ Even though mass cytometry provides unparalleled information on protein expressions at the single cell level, interpreting the high-dimensional data sets is challenging and poses a new computational difficulty, thereby stimulating the development of analysis software.^5,6^

Phenotyping, which entails categorizing a large number of single cells into various cell types based on the expression patterns of cell surface proteins, is one of the most essential tasks in mass cytometry data analysis. There have been two approaches to the analysis of CyTOF data for the phenotyping of heterogeneous immune cell systems: (1) manual gating and (2) unsupervised clustering. To identify cell populations of interest, manual gating consisted of a hierarchical series of visual inspections performed on 2D scatter plots against two cellular markers. The manual gating, however, can be time-consuming and labor-intensive depending on the amount of samples, cellular markers, and cell types. Furthermore, because manual gating is performed only for defined cell types, there are often a large number of unassigned cells remaining after manual gating, which may contain cells of undefined novel categories. In contrast, unsupervised clustering aggregates single cells with comparable expression patterns of cellular markers into an arbitrary number of clusters while simultaneously considering all cellular markers.^7,8^ Many computational methods including FlowSOM,^9^ PhenoGraph,^10^ X-shift,^11^ flowMeans,^12^ SPADE,^13^ SWIFT,^14^ ClusterX,^15^ and Citrus^16^ have been developed for the unsupervised clustering of CyTOF data sets. Unsupervised clustering may identify all cell types, even those that are not yet characterized, while eliminating biases brought on by manual gating. Nevertheless, it is challenging to estimate the actual number of clusters, and clustering algorithms do not scale well with high-throughput, high-dimensional data, such as cohort studies involving hundreds of biological samples.

Both manual gating and unsupervised clustering were hard to reproduce. The manual gating yields different results due to the diversity in the shapes and boundaries of the gates being used depending on the gating strategy employed by the researchers. Unsupervised clustering randomly selects a specified number of cells from all samples to reduce the computational burden; however, such random subsampling may reduce the reproducibility of clustering, especially for rare cell types. To enhance the reproducibility of CyTOF data analysis, semiautomated approaches learned the properties of each cell type from prior knowledge and automatically classified unassigned cells in biologically relevant samples.^17^ Prior knowledge was usually obtained from manual gating of a reference sample. The semiautomated methods have utilized machine learning techniques. DeepCyTOF suggested using a denoising autoencoder and multiple distribution-matching residual networks in a deep learning-based framework.^18^ To overcome variation across samples, DeepCyTOF calibrated input samples to a fixed reference (or training) sample and then performed a domain adaptation procedure for automatic gating. CyAnno utilized various machine learning techniques, including extreme gradient boosting, multilayer perceptron, and support vector machine.^19^ CyAnno proposed a machine learning framework for the integrative modeling of “gated” cell types and “ungated” cells. DGCyTOF combined deep learning-based classification and hierarchical stable-clustering methods to identify predefined and new cell types.^20^ Instead of complex classifiers, a relatively simple LDA classifier based on linear discriminant analysis made it possible to analyze large data sets containing millions of cells.^21^

For semiautomated methods, performance in generalization and ungated cell classification would be crucial. The capacity of a trained machine learning model to generalize to new data is particularly important in supervised learning with a labeled training set. Without adequate generalization, the classifier trained from a single reference might provide erroneous results when applied to different samples, where the expression profiles of cellular markers may be different from those in the reference (due to technical variances between different experimental batches).^22^ Instances from samples that are independent of the reference must be subjected to some kind of domain adaptation technique or fine-tuning in order to have a lower generalization error. Domain adaptation is a collection of techniques that allow a model that has been trained on one data distribution (the source) to be applied to another distribution that is related but distinct (the target). On the one hand, there are typically many ungated cells, or cells without an assigned type, after manual gating. Ungated cells represent a wide range of heterogeneous cell types and are difficult to categorize as a specific cell type with a label because they lack distinguishing characteristics. Most approaches aimed to maximize the performance of gated cells while neglecting ungated cells that were incorrectly classified as gated cell types. As a result, ungated cells are often or almost always misclassified. Ungated cells should not be mistaken for other gated cell types, as they may comprise cells with unknown phenotypes and biological significance, such as immune cells in a transitional state.

In this study, we present CyGate, a semiautomated method for classifying individual cells into predefined cell types or “ungated” cells. Based on a manually gated reference data set, CyGate creates a cell type classifier, which is then used to automatically gate the remaining data sets (from different batches). It is not necessary to manually gate all samples, and machine learning may improve reproducibility by removing analyst’s subjectivity and biases during the phenotyping process. CyGate’s utility was proven by its high classification performance for both predefined and ungated cell types. When compared to other techniques using benchmark data sets, CyGate effectively reduced batch effects between samples and had the lowest generalization error for independent samples.

## Experimental Section

### Data Sets

For the development of the CyGate model and evaluation of the different methods, we employed three publicly available CyTOF data sets that have been used in previous studies for method development: (1) Multicenter study;^23^ (2) Levine;^10^ (3) Samusik.^11^ The multicenter study data set contains 16 CyTOF samples collected at two distinct times with two distinct instruments and consists of four different cell types and eight cellular markers for cell type classification. The Levine data set consists of 14 cell types, with 32 cellular markers from bone marrow cells of two healthy human donors. The Samusik data set consists of 24 cell types with 39 cellular markers from bone marrow cells of 10 different mice. For all data sets, manually gated cell type labels are available in previous publications. The cell populations for each data set are summarized in Table S1. We used manually gated cell type labels as a ground truth to evaluate the performance of the cell type classification.

### Performance Evaluation

We compared CyGate with four semiautomated methods, DeepCyTOF,^18^ CyAnno,^19^ DGCyTOF,^20^ and LDA.^21^ The tool information about all four methods is summarized in Table S2. The different methods were applied to the multicenter study, Levine, and Samusik data sets, all of which are composed of multiple individuals. For each method, we established a cell type classifier using manually gated cell types on a single reference sample and then automatically performed cell type gating on the remaining samples using the learned model. The automated gating results were compared with the manual gating results. The *F*-score was used for the evaluation of the different methods. The *F*-score provides a value between 0 and 1 for each cell type, with 1 indicating a perfect reproduction of the manual gating. The *F*-score for each type is calculated as follows:
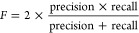
where recall is the fraction of cells retrieved by the prediction algorithm out of all relevant cells, whereas precision is the fraction of relevant cells retrieved out of all cells retrieved by the prediction algorithm. The *F*-score for multiple cell types is calculated as the weighted average of the *F*-scores for each cell type. Let *C* = {*c*_1_, *c*_2_, ..., *c*_*n*_} be a correct set of cell types, |*c*_*i*_| the number of cells in *c*_*i*_, *N* = Σ|*c*_*i*_|. The weighted *F*-score by the number of cells is calculated as follows:
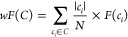


We calculated two *F*-scores based on whether we took into account only cells with manually gated cell types or all cells, including ungated cells, in evaluating the performance of cell type classification. If ungated cells are used for the *F*-score calculation, *C* includes *c*_ungated_, a label for the ungated cell group.

### CyGate Algorithm

**Figure 1:**
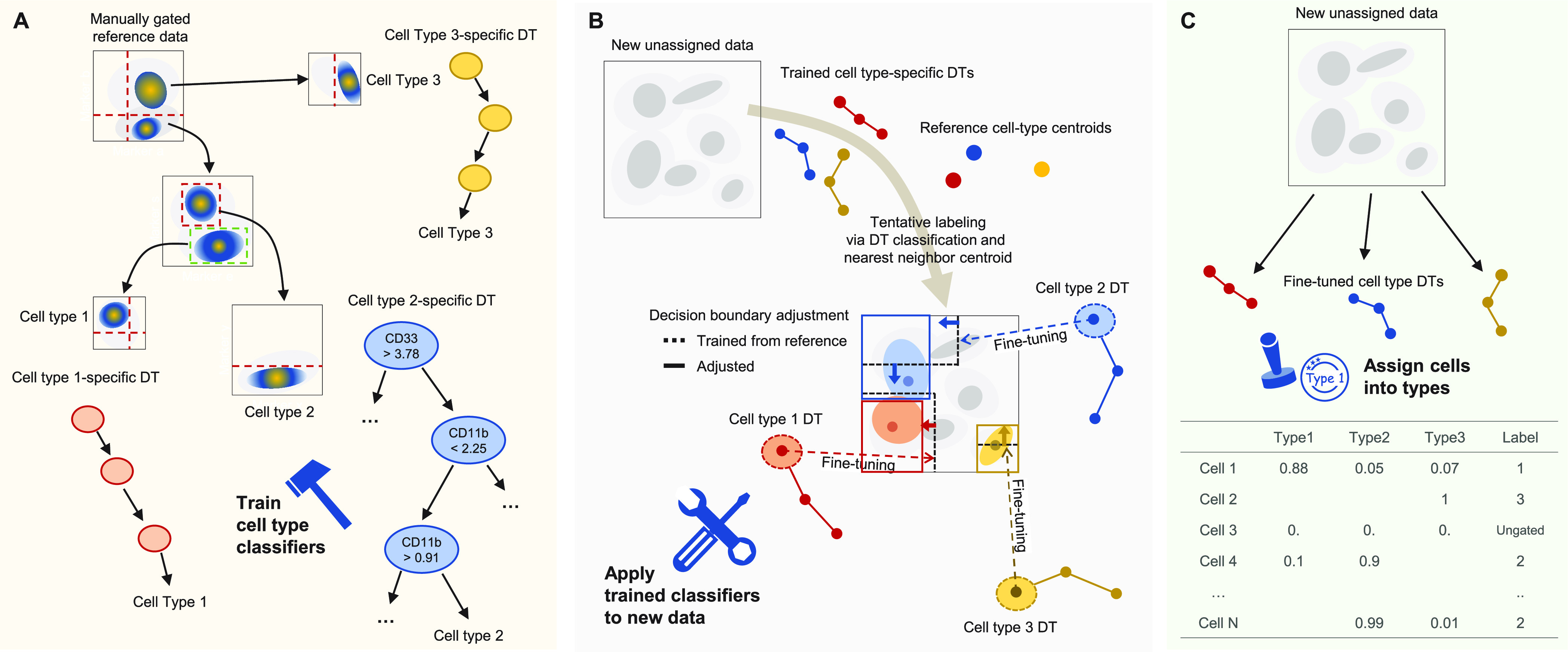
CyGate workflow. (A) Given manually gated reference data for training, cell type-specific classifiers are trained using a decision tree algorithm (DT). (B) Unassigned cells in the input data are tentatively labeled using trained cell-type decision trees and cell-type centroids derived from a reference sample. In the middle box, cells that were labeled as cell types are shown in color, while unlabeled cells are shown in gray. On the basis of the tentative labels, cell type-specific classifiers are optimized for maximum classification. The dashed lines represent conditions of decision trees trained using a reference sample, and the conditions are modified to classify the tentative labels. (C) Unassigned cells are classified using the tuned classifiers.

CyGate takes a reference sample (including cell classification) and unassigned input samples in CSV (comma-separated values) format as inputs. Cellular marker expressions were converted using an arcsinh transformation with a standard cofactor of 5, i.e., arcsinh(*x*/5).^7,16^ The CyGate workflow is summarized in Figure 1. CyGate initially obtained two kinds of information from a reference: cell type centroids and cell type-specific cellular markers (learning step). CyGate employs a decision tree algorithm to train a cell type classifier. By constructing cell type-specific binary decision trees, it is possible to identify cell type-specific cellular markers and their boundaries. CyGate, then, labels unassigned cells in the input data using the cell type decision trees and cell type centroids derived from the reference sample (tentative labeling step). The tentative labels include all predefined cell types and “ungated cells” (see Decision Boundary Tuning for Generalization for details). CyGate modifies the decision conditions of trained binary decision trees in order to more precisely classify tentative cell labels from input data, where the conditions were adjusted only if the introduced positives (from a cell type of interest) were greater than the introduced negatives (from other cell types and ungated cells) (fine-tuning step). Once all fine-tuning tasks for all cell types have been completed, cells can be reclassified based on the updated decision rules (reclassification step). CyGate, unlike other approaches, does not attempt to assign all cells to types; the cells that could not be classified by any of the decision trees are classified as “ungated cells”.

### Cell Type-Specific Decision Trees

To identify cell type-specific cellular markers, CyGate built a decision tree specific to each cell type based on the cell type classification of a reference sample. For the cell types *C* = {*c*_1_, *c*_2_, ..., *c*_*n*_}, each cell type, *c*_*i*_, should have cellular markers distinguishing it from the other cell types. To find such markers, we created two labels, *Target*_*i*_ for *c*_*i*_ of interest and *Other*_*i*_ for {*c*_*k*_ ∈ *C*|*k* ≠ *i*} (i.e., all types other than *c*_*i*_), and built a “one vs rest” binary classifier for the two groups. The two groups are distinguished using a decision tree algorithm, as shown in Figure 1A. A decision tree algorithm constructs a classification model in the form of a tree structure consisting of nodes and branches.^24^ Typically, a decision tree begins with a single root node, which branches into possible outcomes based on the attribute test (marker expression test in this work). These outcomes generate additional nodes, which may, in turn, branch into other possibilities. A leaf node is a node that no longer branches and represents a class label (i.e., a decision made after testing all attributes). With branching, a data set is divided into progressively smaller subsets containing instances with similar characteristics (homogeneous).^24^ Homogeneity of a group is calculated by using entropy. Entropy is zero when a group is completely homogeneous. If the group is heterogeneous (equally divided), then the entropy is 1. Shannon entropy *H* was calculated as follows:
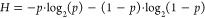
where *p* is the proportion of *Target*_*i*_ at a given node.

When a *parent* node is split into *left* and *right* child nodes, the change in entropy (or information gain) was calculated as follows:
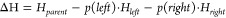
where *p*(*left*) and *p*(*right*) are the proportions of cells in the parent node assigned to each child.

The cellular marker and its condition yielding the greatest reduction in entropy were selected to split a node while growing a decision tree. The depth of a tree was at least two, and branching was terminated when a split node contained less than 90% of *Target*_*i*_ or when the change in entropy was insignificant (entropy reduction, Δ*H*, must be greater than 0.1). Our decision tree involves finding a leaf node that is characterized by at least two cellular markers per cell type and contains more than 90% of *Target*_*i*_. Note that manual gating does not utilize all of the cellular markers to isolate a particular cell type. The decision tree would automatically rediscover the cellular markers and possibly their boundaries used in manual gating, or if better cell separation is available, it might opt for a different set of cellular markers.

### Decision Boundary Tuning for Generalization

CyGate develops a cell classifier using cell type-specific decision trees and employs it to categorize cells from biologically relevant, different samples. We assume that the cellular markers comprising cell type-specific decision trees (i.e., attributes in decision nodes) are not reference sample-specific but rather universal in biologically relevant samples. Consequently, the cellular markers could be consistently applied to the relevant samples. However, the marker boundaries (or marker expression level) may vary from sample to sample due to batch effects, which are data changes produced by nonbiological causes commonly observed in high-throughput experiments.^22^ For precise cell classification, batch effect correction (or marker boundary correction) is required.

The cell classification of new unassigned data set consists of three steps, (1) tentative labeling as *Ĉ* = {*ĉ*_1_, *ĉ*_2_, ..., *ĉ*_*n*_} or *ĉ*_*ungated*_, (2) fine-tuning of trained cell classifiers, and (3) cell classification using fine-tuned classifiers. In the tentative labeling step, unassigned cells were first classified and labeled as *ĉ*_*i*_ using cell type decision trees trained from the reference sample and cells that could not be classified were labeled as *ĉ*_*ungated*_ (Figure 1B). If the batch effects are negligible between training and input samples, the *ĉ*_*i*_ labels are likely to be almost correct. To account for batch effects in reality, *ĉ*_*i*_, a label generated by the initial decision tree, was compared with *ĉ*_*j*_, a label generated by the nearest reference cell type centroid (using Euclidean distance, Ed). When *i* ≠ *j* and Ed(*cell*,*centroid*_*i*_) – Ed(*cell*,*centroid*_*j*_) > *d*_1_, the cell was labeled *ĉ*_*j*_, otherwise, it was *ĉ*_*i*_. The *ĉ*_*ungated*_ cell was labeled *ĉ*_*j*_ if Ed(*cell*,*centroid*_*j*_) < *d*_2_, otherwise it was *ĉ*_*ungated*_. The distance thresholds *d*_1_ and *d*_2_ were empirically decided (*d*_1_ = 1 and *d*_2_ = 0.05 in this work). In the middle box of Figure 1B, cells labeled with cell types are shown in color, while unlabeled cells, *ĉ*_*ungated*_, are shown in gray.

Once tentative labels for all input cells had been determined, cell type-specific decision trees were adjusted to assign more accurate cell labels. Note that one does not need to construct new cell type-specific decision trees for new input data, and one can reuse the structures of the existing decision trees because it is assumed that cellular markers are not reference sample-specific but rather universal in biologically relevant samples. However, the thresholds of nodes (marker conditions) must be re-established because the marker expression levels may change in new input samples. For each cell type-specific decision tree, its associated marker boundaries were searched by tuning them from the cell type centroid computed from the tentative labels; i.e., the cell type centroid is assumed to be within the boundaries (we observed that the tentative cell type centroids were very close to the real centroids, except for ungated cells). The boundary tuning is stopped if the introduced positives (from a cell type of interest) are less than the introduced negatives (from the other types and ungated cells) or if the introduced population is small (less than 1%) relative to the population at the densest point within the boundaries. Once the marker boundaries have been fixed for all cell types, cells can be reclassified based on the updated marker boundaries. The cells that could not be assigned by any of the decision trees are classified as “ungated cells”. Certain cellular markers are shared by multiple cell types; for instance, “CD11bhi monocyte cells” and “CD11bmid monocyte cells” are identified by their CD11b expression. Some cells may be assigned multiple types during reclassification using adapted boundaries, because cell type-specific decision trees are independent of one another. A cell with multiple types is assigned to the type with the lowest level of impurity in terms of tentative labels (Figure 1C). After assignment of cell types to all cells in an input sample, the cell type centroids of the assigned cells are newly calculated and compared to those of a reference sample. If the majority of new centroids deviated from reference centroids, it is assumed that there were batch effects between the input and reference samples; the classification procedure is then repeated by using the new centroids and fine-tuned decision trees in place of those from the reference sample.

### Software Availability

CyGate was developed using the Java programming language and can be accessed at https://github.com/seungjinna/cygate.

## Results and Discussion

### Manual Gating and Machine Learning

Manual gating is carried out by drawing gate boundaries on the plane of two markers to identify cell populations of interest. The cells that lie within the gate boundaries are subjected to further selection, whereas the cells that remain outside the boundaries, i.e., ungated cells, are discarded. This manual gating process closely resembles a decision tree utilized in machine learning approaches. We modified a decision tree algorithm to imitate the manual gating procedure so that the ungated cells can remain intact. Our algorithm does not simultaneously categorize all cell types in a single tree. Instead, it builds cell type-specific, “one vs rest” binary decision trees, where one group represents a cell type of interest and the other group is the remainder (see the Experimental Section for details). We could classify each cell type rapidly using the corresponding decision tree. “Ungated cells” are cells that do not belong to a specific cell type.

We argue that it is advantageous to use cell type-specific binary decision trees rather than a global multiclass tree that classifies all cell types. In a global multiclass tree, it is difficult to determine which node is associated with a particular cell type. In a root node, for instance, the condition is specific to cell type 1 but not to cell type 2. It is complicated to fine-tune the global decision tree because a change in a node condition affects multiple leaf nodes (i.e., multiple cell types), and the reclassification results in all leaf nodes must be evaluated. In contrast, in a cell type-specific decision tree, all node conditions must be specific to the given cell type, and only a leaf node, which includes cells of interest, is involved in classifying the cell type. Note that cells of a particular cell type are clustered because their expression patterns of cellular markers are basically identical (similar in reality). Due to this property, cells of a particular cell type are not classified into multiple leaf nodes of the cell type-specific decision tree but only into one, which is an important benefit of the cell type-specific decision tree. To fine-tune a cell type-specific decision tree for new data, one can modify the conditions along the path from the root node to the leaf node (i.e., decision rule) and evaluate the reclassification results in the leaf node. It is relatively simple to fine-tune the conditions because the conditions along the path form a conjunction, and the number of conditions (associated with one cell type) is small (normally less than 5).

### Generalization on Biologically Relevant Samples

**Figure 2:**
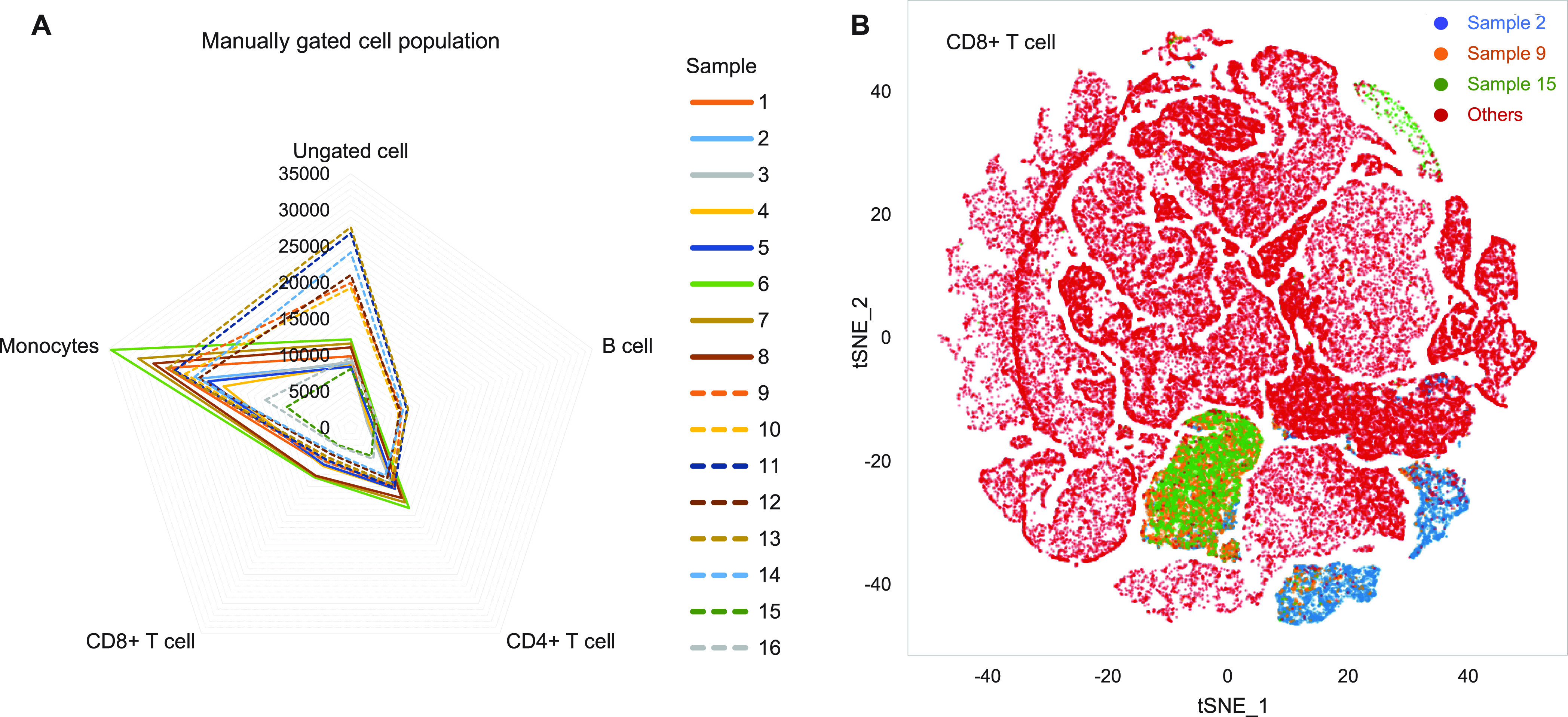
(A) Cell populations of the multicenter data set. The data comprised eight-dimensional markers for four cell types. (B) t-SNE plot of the cells in samples 2, 9, and 15. The CD8+ T cells in samples 2, 9, and 15 are represented in blue, orange, and green, respectively.

Generalization in machine learning is the ability of a trained model to adapt to new input, particularly in supervised learning that uses a labeled training set. This capability is especially crucial for semiautomated gating approaches that analyze new samples independently of training data. We highlight the importance of generalization using multicenter data consisting of 16 samples from the same subject. In multicenter data, the first eight samples were collected simultaneously and analyzed using the same instrument, while the remaining eight samples were obtained two months later and analyzed using a different instrument.^23^Figure 2A depicts the cell populations of the 16 samples, including ungated cells (dashed line for the last eight samples). The t-SNE embedding of the cells in samples 2, 9, and 15 in Figure 2B suggests that the varied settings may result in batch effects. Even cells of the same type are distributed differently on the t-SNE map; therefore, applying a trained classifier uniformly to all data could result in an erroneous cell classification. For example, the *F*-score was 0.994 when 25% of the cells from sample 2 were used to train a cell type classifier, and the cell types of the remaining 75% of the cells were predicted. In contrast, when a cell type classifier trained using all cells from sample 2 was applied to samples 9 and 15 to classify cells, the resulting *F*-scores were 0.86 and 0.62, respectively. For accurate classification, the adaptation between training samples and new samples or the tuning of a classifier to a new sample could be advantageous. DeepCyTOF proposed the adaptation of new samples to a training sample.^18^

**Figure 3:**
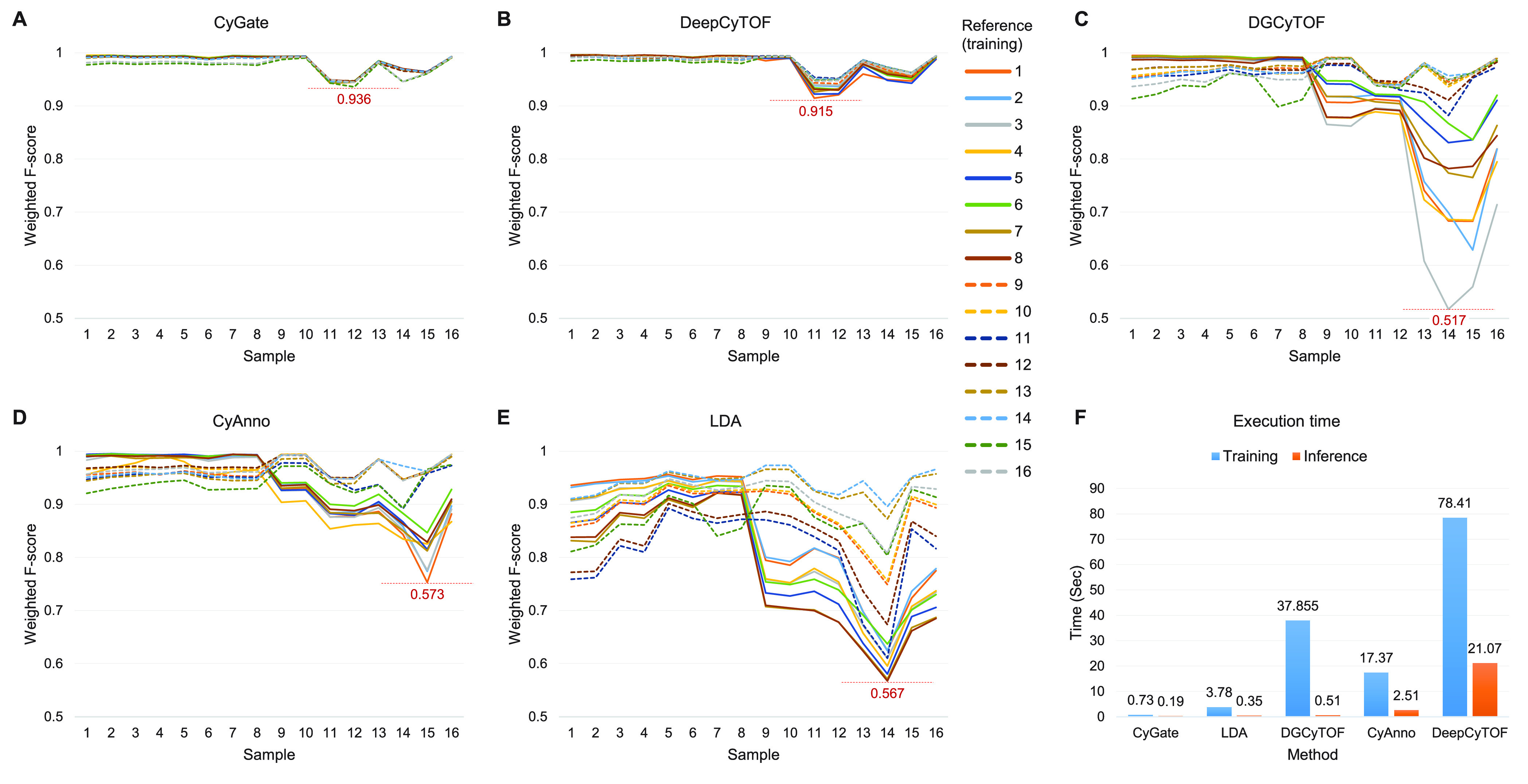
Cell classification performance of (A) CyGate, (B) DeepCyTOF, (C) DGCyTOF, (D) CyAnno, and (E) LDA on the multicenter data set. Each sample was chosen as a reference for training a cell classifier, resulting in 16 distinct classifiers (shown in different colors). Each classifier was applied to all samples to classify cells, and its classification performance for manually gated cell types is shown as weighted *F*-scores. Dashed lines show the *F*-scores of classifiers trained with samples 9 through 16. (F) The training (blue) and inference (orange) times of different methods are shown. The methods were executed on a server equipped with an Intel Xeon Scalable CPU (6248R, 24 cores, 3.0 GHz).

We assessed the performance of semiautomated gating methods such as DeepCyTOF,^18^ CyAnno,^19^ DGCyTOF,^20^ and LDA^21^ and compared them with CyGate’s results. Each method was initially trained on a reference sample before it was applied to the remaining samples. Each sample in the multicenter data set was chosen as a reference, resulting in 16 different classifiers that were applied to all samples, including the reference sample, to classify cells. Figure 3 depicts performance in terms of *F*-scores (averages weighted by population size), which were calculated only on manually gated cells (ungated cells were not accounted for). CyGate has the highest average *F*-score when compared to other methods, and its superiority in generalization is demonstrated by the lowest variance in *F*-scores. The color in Figure 3 represents a model trained using a reference sample. It is noteworthy that the performance of CyGate is hardly dependent on the training sample; the fact that multicenter data were collected under two settings is not revealed. In contrast, the DGCyTOF and LDA performances in Figure 3C,E are very unstable for sample 14 and heavily dependent on the training sample. The averages weighted by population size give more importance to relatively larger populations. Unweighted *F*-scores are shown in Figure S1 so that cell types of both large and small populations are given equal representation. In addition, we compared the average training and inference times of various approaches in Figure 3F, which varied by several orders of magnitude. The training time was quantified as the duration required to train a cell classifier using a manually gated reference sample, and the inference time was quantified as the duration required for the trained classifier to assign cell types to all cells in an input sample. CyGate’s execution time was the shortest for both training and inference, followed by LDA.

### Ungated Cell Classification

**Figure 4:**
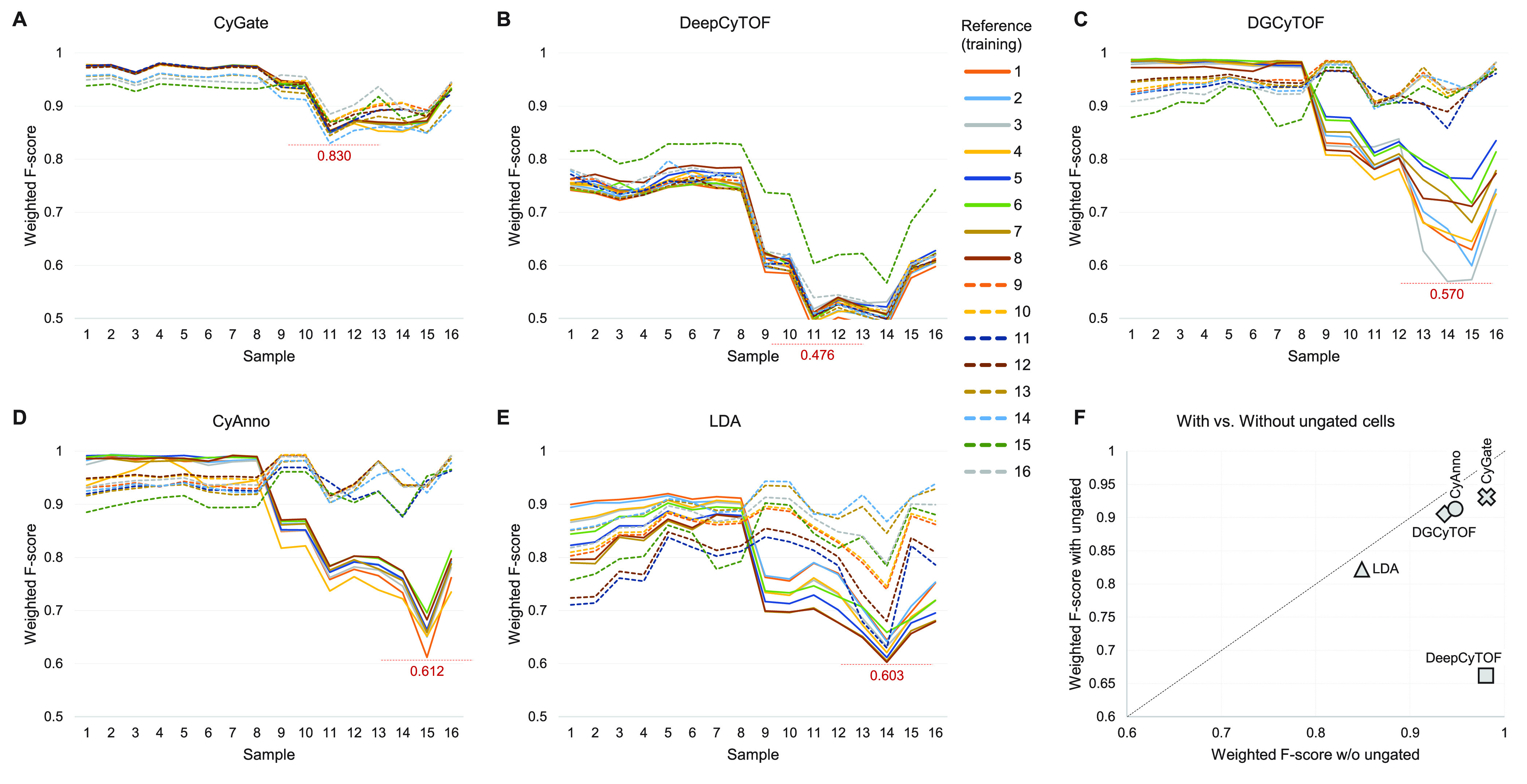
Cell classification performance of (A) CyGate, (B) DeepCyTOF, (C) DGCyTOF, (D) CyAnno, and (E) LDA on the multicenter data set when ungated cells are considered as a cell type label. Each sample was chosen as a reference for training a cell classifier, resulting in 16 distinct classifiers (shown in different colors). Each classifier was applied to all samples to classify cells, and its classification performance is shown as weighted *F*-scores. Dashed lines show the *F*-scores of classifiers trained with samples 9 through 16. (F) The average *F*-scores are compared when ungated cells are considered (*y*-axis) versus when they are not (*x*-axis).

After manual gating, there are often a substantial number of ungated cells. Although ungated cells may have unknown phenotypes and biological significance, there is no consensus about how to handle them. Most computational approaches, including clustering methods, have been applied to all cells (including ungated cells), but only manually gated cells have been used to evaluate their performance. As a result, ungated cells are often misclassified as gated cell types. If categorization of ungated cells into known cell types can be avoided, it can be helpful in identifying unknown and rare cell types. We evaluated the efficacy of various methods for classifying ungated cells as a particular cell type. Figure 4 illustrates *F*-scores (averages weighted by population size) when ungated cells are included in the cell classification performance (unweighted *F*-scores are shown in Figure S2). CyGate not only has the highest *F*-score average but also shows the lowest variance in *F*-scores among the four methods. In contrast, the performances of DGCyTOF, CyAnno, and LDA are heavily dependent on training data, showing that the multicenter data were collected under two distinct settings. In Figure 4C,D, the models trained using the first eight samples performed well on the first eight samples but poorly on the last eight samples (solid lines) and vice versa. Figure 4F shows that *F*-scores decreased for all methods when ungated cells were considered, indicating that classification of ungated cells is a challenge. DeepCyTOF’s *F*-score decreased dramatically as the bulk of ungated cells were misclassified as gated cell types.

### Application to Complex Data Sets

**Figure 5:**
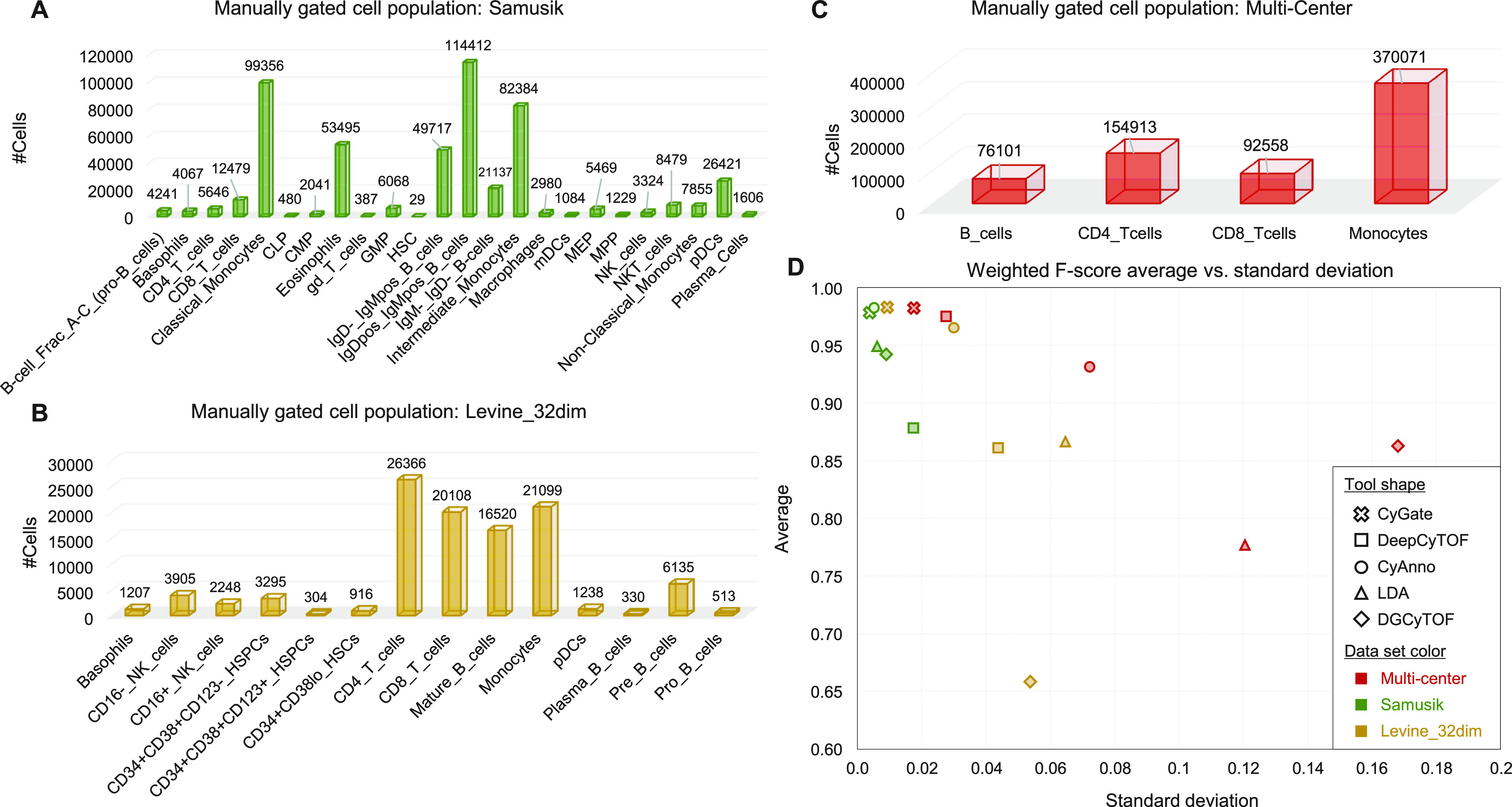
Application to three CyTOF data sets: Samusik, Levine, and multicenter. Manually gated cell populations are shown for (A) Samusik, (B) Levine, and (C) multicenter data sets. (D) The average *F*-score (*y*-axis) and standard deviation (*x*-axis) are displayed for each data set. The values were computed using the *F*-scores for each sample when each method classified cells from all samples. *F*-scores were calculated only on manually gated cells. Different shapes and colors correspond to distinct data sets and methods, respectively.

CyGate was applied to two complex CyTOF data sets with dozens of cellular markers: (1) Levine data set consisting of 14 cell types with 32 cellular markers from bone marrow cells of two healthy human donors;^10^ (2) Samusik data set consisting of 24 cell types with 39 cellular markers from bone marrow cells of 10 different mice.^11^ These two data sets, like the multicenter data set, consisted of multiple individuals and experiments were conducted in the same settings, respectively. These two data sets differ from the multicenter data set in that the batch effects are negligible, as the barcoding technique was adopted to measure single cells from multiple individuals in a single batch.^25^ The number of cells per cell type is shown in Figure 5. The cell types and populations vary from data set to data set, and Samusik (Figure 5A) and Levine (Figure 5B) data sets contain rare cell types, such as “HSC” and “gd T cells” in Samusik and “CD34+CD38+CD123+HSPCs” and “Plasma B cells” in Levine.

To compare the generalization performance of methods with and without batch effects, we computed the standard deviation of *F*-scores, where a larger standard deviation indicates a poorer generalization performance. When cell classifiers trained on distinct reference samples were applied to all samples, the classifier with the greatest standard deviation in *F*-scores was chosen for performance comparison. Figure 5D depicts its average *F*-score and standard deviation. When classifying the Samusik data set, the standard deviations were small for all methods (shown in green in Figure 5), whereas they grew larger for the multicenter data set (shown in red). It is because the Samusik data set was measured in a single batch, while the multicenter data set was measured in two different batches. CyAnno appears susceptible to batch effects (O-shapes in Figure 5); it performed well on the Samusik data set but not on the other data sets. CyGate maintained relatively small standard deviations as well as high *F*-scores across all data sets (X-shapes in Figure 5), demonstrating that its generalization is superior to that of other methods. The weighted *F*-scores may be dominated by major cell populations such as “IgDpos IgMpos B cells” and “Classical Monocytes” in the Samusik data set; therefore, the performances were also compared by using unweighted *F*-scores. CyGate consistently exhibited the best performance when compared using unweighted *F*-scores (Figure S3), indicating that it can effectively classify rare cell types as well.

## Conclusions

Instead of time-consuming manual gating, CyGate learns a gating strategy from a single (manually gated) reference sample, trains a model for cell classification, and then analyzes new samples automatically using the trained model. CyGate eradicated the subjectivity of operators in manual gating, where results could vary from operator to operator and substantially improved the consistency of gating analyses. CyGate is the quickest semiautomatic gating method available. Due to its explainability, ease of implementation, and low training time, the decision tree method is simple and easy to use, compared to cutting-edge machine learning techniques, such as deep learning. Moreover, CyGate established cell type-specific classifiers, making it easy to adapt the classifiers to new data and achieve excellent generalization. CyGate’s performance in classifying cells was unaffected by batch effects between training and new data sets, whereas the performance of other tools decreased significantly (Figure 4).

CyGate provides a machine learning framework for classifying ungated cells, enabling the potential discovery of rare, unknown cell types through further investigation. CyTOF enables the measurement of hundreds of thousands of single cells per sample, but the cell types of some of these cells remain unknown. Typically, such ungated cells constitute a significant proportion of all measured cells: 25% of multicenter data, 61% of Samusik data, and 38% of Levine data (Table S1). Ungated cells may contain cells with unknown phenotypes, but the designed cellular markers may not be able to differentiate them. Many approaches have confused ungated cells with other gated cell types.

CyGate would ultimately enhance interinstitutional collaboration by facilitating it. CyGate exhibited its outstanding capacity for generalization in tests utilizing benchmark data sets by producing consistent gating results for samples obtained under conditions distinct from those for training sample collection. If just a manually gated reference sample is shared with collaborators, then CyGate will assess samples collected from various locations using a consistent gating strategy with high reliability.

## Supporting Information Available

Table S1, Manually gated cell population; Table S2, Software resources: DeepCyTOF, CyAnno, DGCyTOF, and LDA; Figure S1, Performance comparisons using unweighted *F*-scores; Figure S2, Performance comparisons using unweighted *F*-scores when ungated cells are considered; Figure S3, Application to three CyTOF data sets: Samusik, Levine, and multicenter (PDF)
