# Supplementary material for: CyGate Provides a Robust Solution for Automatic Gating of Single Cell Cytometry Data
Source: Anal Chem. 2023 Nov 9;95(46):16918–26. doi: 10.1021/acs.analchem.3c03006 (PMC10666088; doi:10.1021/acs.analchem.3c03006)
Supplement: Supplementary file 1 — ac3c03006_si_001.pdf [file ac3c03006_si_001.pdf]

Supporting Information

CyGate provides a robust solution for automatic gating of single cell cytometry data

Seungjin Na[1,2], Yujin Choo[3], Tae Hyun Yoon[4,5,6], and Eunok Paek[1,2,3*]

[1]Institute for Artificial Intelligence Research, Hanyang University, Seoul 04763, Republic of Korea,
[2]Dept. of Computer Science, Hanyang University, Seoul 04763, Republic of Korea,
[3]Dept. of Artificial Intelligence, Hanyang University, Seoul 04763, Republic of Korea,
[4]Dept. of Chemistry, College of Natural Sciences, Hanyang University, Seoul 04763, Republic of Korea,
[5]Institute of Next Generation Material Design, Hanyang University, Seoul 04763, Republic of Korea,
[6]Yoon Idea Lab Co., Ltd., Seoul 04763, Republic of Korea

Table of Contents

Table S1. Manually gated cell population.

Table S2. Software resources: DeepCyTOF, CyAnno, DGCyTOF, and LDA.

Figure S1. Performance comparisons using unweighted F-scores.

Figure S2. Performance comparisons using unweighted F-scores when ungated cells are considered.

Figure S3. Application to three CyTOF data sets: Samusik, Levine, and multi-center.

Table S1. Manually gated cell population.

A

Manually gated cell population: Multi-Center 16 samples																	
Cell type	1	2	3	4	5	6	7	8	9	10	11	12	13	14	15	16	Sum
B_cells	2503	2384	2337	2583	2963	3740	3377	3472	7385	6926	8153	7166	8325	7568	3470	3749	76101
CD4_Tcells	10329	9455	9556	10460	10423	13682	12756	11991	10060	9536	10239	8594	9566	8113	4836	5317	154913
CD8_Tcells	6305	5877	5918	6522	6308	8454	8269	8176	5783	5454	5534	4555	5110	4214	2982	3097	92558
Monocytes	26656	21854	20701	18408	20606	34715	30754	28553	25695	23989	25462	21985	26481	22622	9246	12344	370071
Ungated	9828	8782	9069	8597	8413	12171	11610	11066	19965	19272	26775	21017	27612	24112	8103	9650	236042

B

Manually gated cell population: Samusik, 10 samples											
Cell type	1	2	3	4	5	6	7	8	9	10	Sum
B-cell_Frac_A-C_(pro-B_cells)	266	473	344	477	480	422	510	446	480	343	4241
Basophils	292	409	368	566	437	432	407	404	450	302	4067
CD4_T_cells	406	526	579	722	583	423	819	573	534	481	5646
CD8_T_cells	798	1248	1323	1595	1078	941	1590	1520	1245	1141	12479
Classical_Monocytes	13607	12083	11762	9630	8403	10092	9302	8781	11152	4544	99356
CLP	59	56	52	34	35	39	51	46	46	62	480
CMP	197	203	199	229	278	196	232	173	206	128	2041
Eosinophils	5173	6233	4650	7763	4816	4804	4554	6246	4911	4345	53495
gd_T_cells	35	38	32	40	39	41	56	38	26	42	387
GMP	703	722	599	762	583	661	527	430	650	431	6068
HSC	3	2	1	3	1	2	2	3	5	7	29
IgD-_IgMpos_B_cells	3508	4841	4296	5070	5115	3848	6480	6087	5717	4755	49717
IgDpos_IgMpos_B_cells	8768	13919	14439	8975	11444	8311	14673	11840	11677	10366	114412
IgM-_IgD-_B-cells	1123	1850	1607	1981	2460	2267	2957	2720	2389	1783	21137
Intermediate_Monocytes	12045	7827	8629	7013	8107	8154	7662	6689	6935	9323	82384
Macrophages	199	178	273	386	342	441	215	343	330	273	2980
mDCs	116	118	112	95	143	95	104	104	129	68	1084
MEP	420	566	490	682	592	584	618	495	584	438	5469
MPP	197	129	109	129	129	92	116	108	104	116	1229
NK_cells	189	282	272	412	445	408	346	246	533	191	3324
NKT_cells	961	947	807	925	775	782	715	839	908	820	8479
Non-Classical_Monocytes	1016	1024	913	766	538	402	817	778	718	883	7855
pDCs	2974	2542	2639	2810	2478	2361	2729	2805	2617	2466	26421
Plasma_Cells	118	163	353	61	464	41	195	31	108	72	1606
Ungated	33691	31393	29708	29587	34845	31443	39410	33996	31052	32133	327258

C

Manually gated cell population: Levine_32dim, 2 samples			
Cell type	1	2	Sum
Basophils	946	261	1207
CD16-_NK_cells	2602	1303	3905
CD16+_NK_cells	1778	470	2248
CD34+CD38+CD123-_HSPCs	2066	1229	3295
CD34+CD38+CD123+_HSPCs	182	122	304
CD34+CD38lo_HSCs	666	250	916
CD4_T_cells	18131	8235	26366
CD8_T_cells	13205	6903	20108
Mature_B_cells	12320	4200	16520
Monocytes	16111	4988	21099
pDCs	658	580	1238
Plasma_B_cells	243	87	330
Pre_B_cells	3352	2783	6135
Pro_B_cells	203	310	513
Ungated	118888	42555	161443

Table S1. Manually gated cell population. The numbers of cells per cell type are shown for (A) multi-center, (B) Samusik, and Levine_32dim data sets. The multi-center study data set contains 16 CyTOF samples collected at two distinct times with two distinct instruments and consists of four different cell types and eight cellular markers for classifying cell types. The Levine data set consists of 14 cell types with 32 cellular markers from bone marrow cells of two healthy human donors. The Samusik data set consists of 24 cell types with 39 cellular markers from bone marrow cells of 10 different mice.

Table S2. Software resources: DeepCyTOF, CyAnno, DGCyTOF, and LDA.

Software	Web site	Implementation	Reference
DeepCYTOF	https://github.com/lkshan/DeepCYTOF-with-Synthetic-gradient	python	1
CyAnno	https://github.com/abbioinfo/CyAnno	python	2
DGCyTOF	https://github.com/lijcheng12/DGCyTOF	python	3
LDA	https://github.com/tabdelaal/CyTOF-Linear-Classifier	R	4

Reference

(1) Li, H.; Shaham, U.; Stanton, K. P.; Yao, Y.; Montgomery, R. R.; Kluger, Y. Gating mass cytometry data by deep learning. *Bioinformatics* **2017**, 33, 3423–3430.

(2) Kaushik, A.; Dunham, D.; He, Z.; Manohar, M.; Desai, M.; Nadeau, K. C.; Andorf, S. CyAnno: A semi-automated approach for cell type annotation of mass cytometry datasets. *Bioinformatics* **2021**, 37, 4164–4171.

(3) Cheng, L.; Karkhanis, P.; Gokbag, B.; Liu, Y.; Li, L. DGCyTOF: Deep learning with graphic cluster visualization to predict cell types of single cell mass cytometry data. *PLoS Comput. Biol.* **2022**, 18, e1008885.

(4) Abdelaal, T.; van Unen, V.; Höllt, T.; Koning, F.; Reinders, M. J. T.; Mahfouz, A. Predicting Cell Populations in Single Cell Mass Cytometry Data. *Cytom. Part A* **2019**, 95, 769–781.

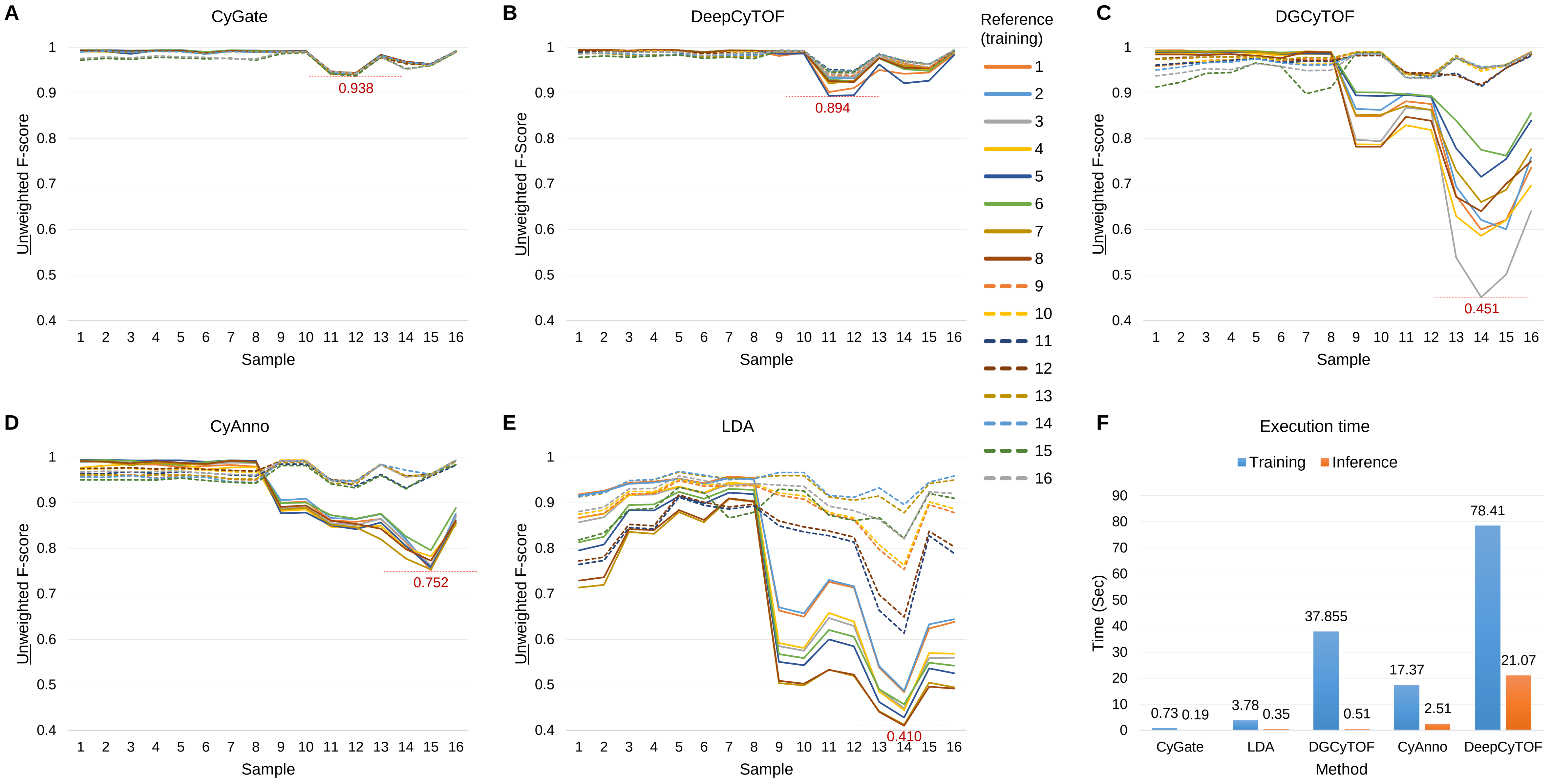

Figure S1. Performance comparisons using unweighted F-scores. Cell classification performance of (A) CyGate, (B) DeepCyTOF, (C) DGCyTOF, (D) CyAnno and (E) LDA on the multi-center data set. Each sample was chosen as a reference for training a cell classifier, resulting in 16 distinct classifiers (shown in different colors). Each classifier was applied to all samples to classify cells, and its classification performance for manually gated cell types is shown as unweighted F-scores (not weighted by population size). Dashed lines show the F-scores of classifiers trained with samples 9 through 16. (F) The training (blue) and inference (orange) times of different methods are shown. The methods were executed on a server equipped with an Intel Xeon Scalable CPU (6248R, 24 cores, 3.0 GHz).

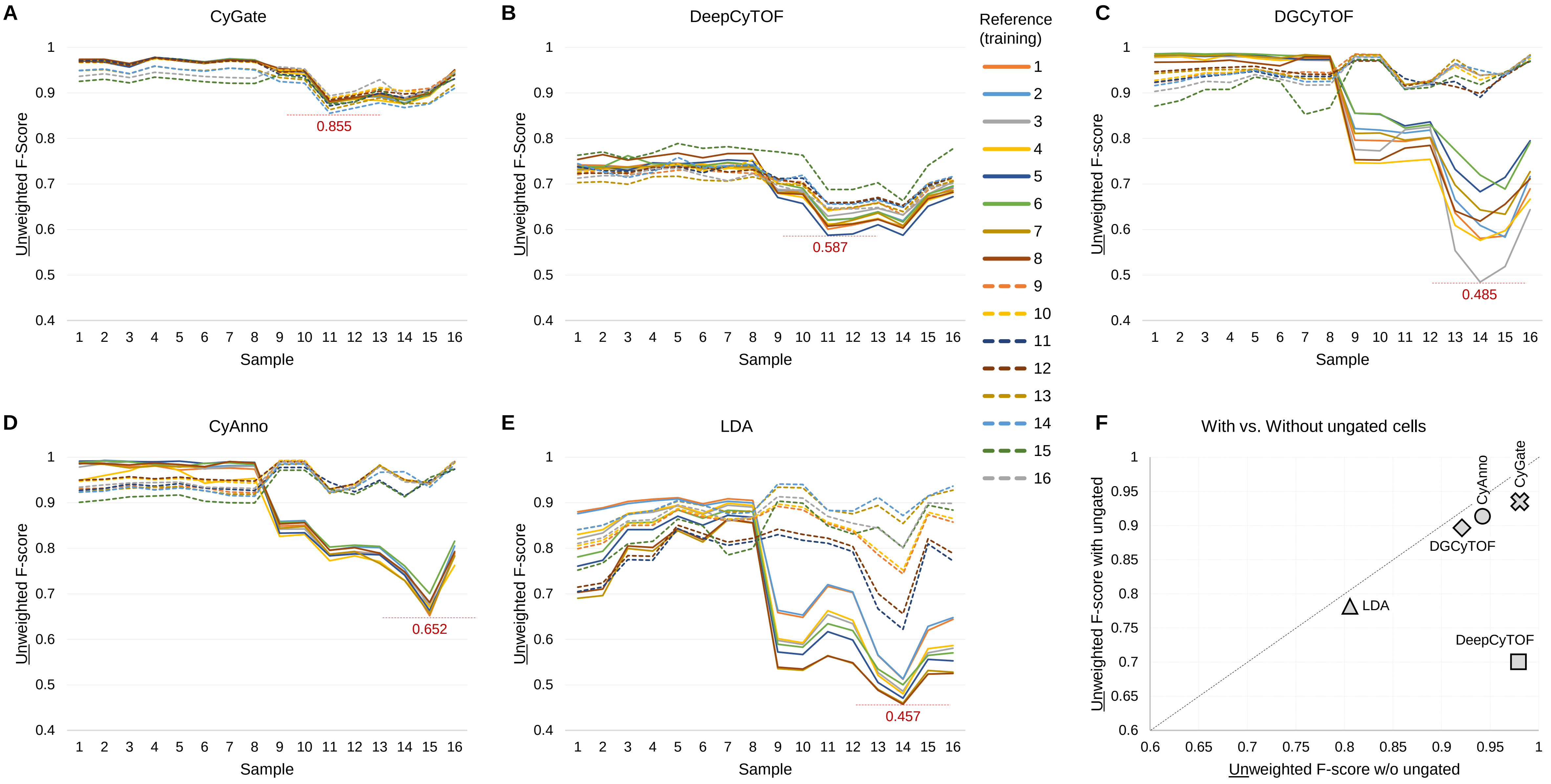

Figure S2. Performance comparisons using unweighted F-scores when ungated cells are considered. Cell classification performance of (A) CyGate, (B) DeepCyTOF, (C) DGCyTOF, (D) CyAnno, and (E) LDA on the multi-center data set when ungated cells are considered as a cell type label. Each sample was chosen as a reference for training a cell classifier, resulting in 16 distinct classifiers (shown in different colors). Each classifier was applied to all samples to classify cells, and its classification performance is shown as weighted F-scores (not weighted by population size). Dashed lines show the F-scores of classifiers trained with samples 9 through 16. (F) The average F-scores are compared when ungated cells are considered (y-axis) versus when they are not (x-axis).

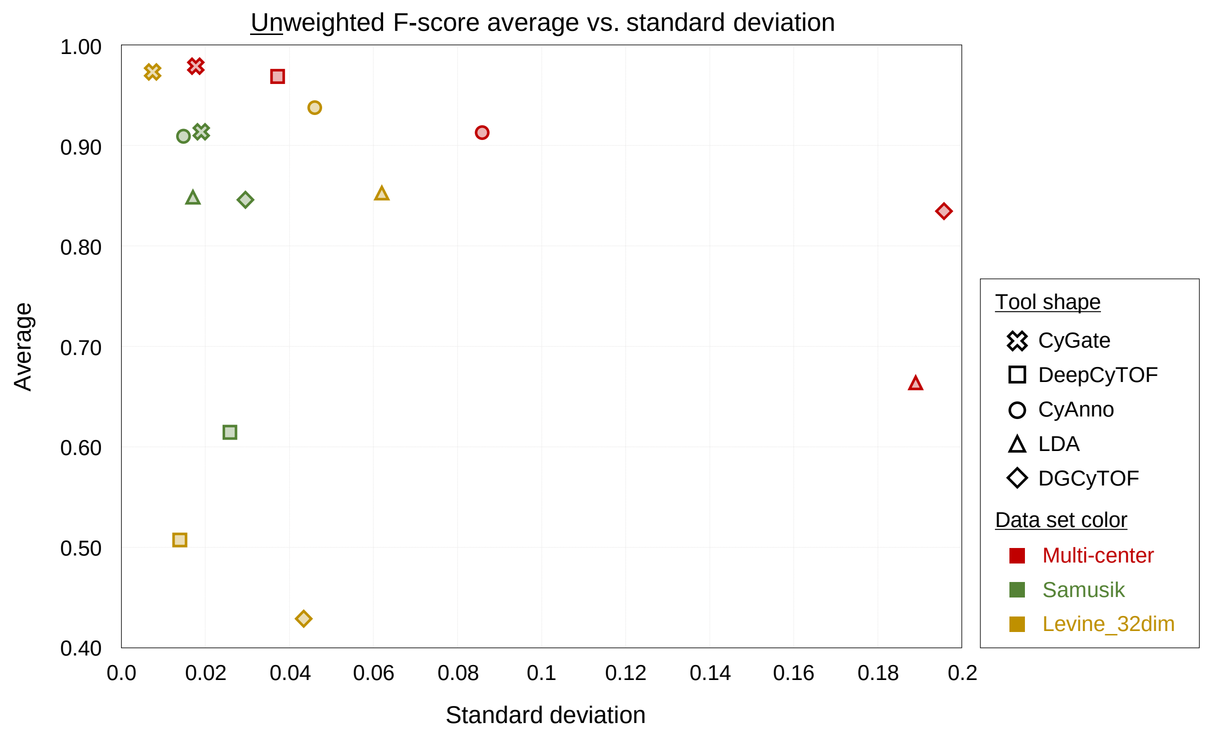

Figure S3. Application to three CyTOF data sets: Samusik, Levine, and multi-center. The unweighted average F-score (y-axis) and standard deviation (x-axis) are displayed for each data set. The values were computed using the F-scores for each sample when each method classified cells from all samples. F-scores were calculated only on manually gated cells and not weighted by population size. Different shapes and colors correspond to distinct data sets and methods, respectively.
